# Western Academy of Natural Sciences

**Published:** 1835

**Authors:** 


### III. — Western Academy of Natural Sciences.

An association of gentlemen in this city, under the above title, has recently been formed, for the purpose of studying and developing the natural history and resources of those parts of the Valley of the Mississippi, to which they may be able to extend their researches. Knowing them as we do, personally, we are enabled to say that among them there are several who, by their attainments and zeal, are well prepared for the important work of science and patriotism which lies before them. They have opened an eligible apartment in which to prepare and arrange their collection, which is designed to embrace specimens of animals, plants and minerals, not a few of which have already been brought together. They have, also, begun the formation of a library of natural science, and will be particularly gratified to receive dissertations, tracts and even newspaper notices, on the zoology, botany, mineralogy, geology, and meteorology of the West, from the Lakes to the Gulf of Mexico. Specimens of its natural productions are, also, respectfully solicited; and whenever the donor desires it, he will be furnished with a correct account of the names of the specimens he may send, which, if he have kept duplicates, will at once give him the advantage of the labours of the Academy.

The officers for the present year are:

Robert Buchanan, *President.*
John P. Foote, Isaac Colby, } *Vice Presidents.*
George Graham, *Corresponding Secretary.*
William Wood, *Recording Secretary.*
James Smith Armstrong, *Treasurer.*
Robert Buchanan, John Locke, Joseph Dorfueille, James H. Perkins, John L. Riddell, } *Curators.*

Boxes of minerals, shells, fossils, bones, and other specimens may be sent, at the expense of the Academy, to the care of its president, Robert Buchanan, Cincinnati. A notice of its locality should always accompany each specimen.

